# Combination of Real-Value Smell and Metaphor Expression Aids Yeast Detection

**DOI:** 10.1371/journal.pone.0007939

**Published:** 2009-11-20

**Authors:** Kouki Fujioka, Eiji Arakawa, Jun-ichi Kita, Yoshihiro Aoyama, Toshiro Okuda, Yoshinobu Manome, Kenji Yamamoto

**Affiliations:** 1 International Clinical Research Centre, Research Institute, International Medical Centre of Japan, Tokyo, Japan; 2 Institute of DNA Medicine, Research Centre for Medical Sciences, Jikei University School of Medicine, Tokyo, Japan; 3 Department of Bacteriology, National Institute of Infectious Diseases, Tokyo, Japan; 4 Research and Development Group, Business Development Department, Analytical and Measuring Instruments Division, Shimadzu Corporation, Kyoto, Japan; 5 Sanwa Norin Corporation, Ltd., Saitama, Japan; Massachusetts Institute of Technology, United States of America

## Abstract

**Background:**

Smell provides important information about the quality of food and drink. Most well-known for their expertise in wine tasting, sommeliers sniff out the aroma of wine and describe them using beautiful metaphors. In contrast, electronic noses, devices that mimic our olfactory recognition system, also detect smells using their sensors but describe them using electronic signals. These devices have been used to judge the freshness of food or detect the presence of pathogenic microorganisms. However, unlike information from gas chromatography, it is difficult to compare odour information collected by these devices because they are made for smelling specific smells and their data are relative intensities.

**Methodology:**

Here, we demonstrate the use of an absolute-value description method using known smell metaphors, and early detection of yeast using the method.

**Conclusions:**

This technique may help distinguishing microbial-contamination of food products earlier, or improvement of the food-product qualities.

## Introduction

Since the deterioration and quality variation of food and drink are often associated with microbial activity, several previous reports have attempted to analyze the smells produced by different microorganisms. They used gas chromatography (GC)/mass spectrometry (MS) techniques to detect and discriminate between these scents [1], [2]. While GC is useful for accurately identifying these odours, the large amount of detailed information generated is too difficult to analyze on a routine basis. Since the ‘electronic nose’ offers a more simple solution for gaining specific odour information, these devices have attracted considerable attention [3]. The devices electronically mimic the mammalian olfactory system [4] in which several olfactory receptors respond to smells [5], [6], [7], [8]. Electronic noses employ several sensors in place of these receptors. These sensors include conductive polymers [9], [10], semiconductors [4], [11], [12] and metalloporphyrins [13].

Research has tested the validity of using electronic noses in a variety of applications [14]. These primarily include testing the quality of food and drink [3], as well as the direct detection of microorganisms [15], [16]. In the former situation, electronic noses were used to estimate the freshness of meat [17] and fish [18], or to discriminate the quality of milk [19]. In the latter case, these devices were used to test for organisms associated with spoilage, including bread moulds [9] and anaerobic bacteria [16]. All the information can be used to check the quality and thereby may aid in preventing intoxication.

However, two important obstacles still prevent the routine use of electronic nose measurements: 1. we cannot identify what differences there are between smells, and 2. we cannot compare and assemble the data collected between different electronic noses. The reason behind these obstacles stems from the fact that the data generated by different electronic noses are sensor specific. Moreover, current electronic noses are designed to detect certain smells using different sensors. To understand the difference and to discriminate between various smells, should we prepare many sensors against possible smells?

To solve this problem even in cases using a small number of sensors, we propose using a new smell description method that combines smell intensity and smell specifications analysis, akin to how sommeliers describe the aroma of wine.

First, in order to express the criteria of smell intensity as an absolute value, we propose the development of a standard odour index. The odour index concept in this study was originally introduced in the Japanese Offensive Odour Control Law (1971) and has been used as a means to measure environmental odours. It includes information on how much a smell sample can be diluted to reach the human nose threshold, the lowest concentration which human noses can detect. The odour index is defined as 10·log_10_ (dilution rate). This formula was derived by the careful analysis of the human olfactory recognition system [20]. It has been shown that humans can not differentiate the intensities of smells by their liner concentrations. The index 10 implies that most humans can detect smells in 10-fold dilutions, and an index of 20 indicates a 100-fold dilution. Using this index, odour from chewing gum were set around 40 and restrooms odours were set around 30 in our previous study [21]. These odour indices help us record and imagine smell intensities as absolute values.

Our second consideration was the criteria of smell specification. We proposed describing this aspect of odour using known smell categories provided by standard gasses (metaphor expression). Using the known smell information provided by standard gases, the amount of accessible information we can use for describing smells can increase dramatically. There are, for example, two common ways to describe the flavour of tea. ‘This flavour is sweet’ and ‘This flavour is like muscat’; the latter metaphor description enables us to imagine the flavour better.

We enabled an electronic nose, FF-2A (Shimadzu Corporation, Japan) [21], [22], to report on both intensity and specification. The device, therefore, can calculate a virtual odour index in terms of standard gas categories.

**Figure 1:**
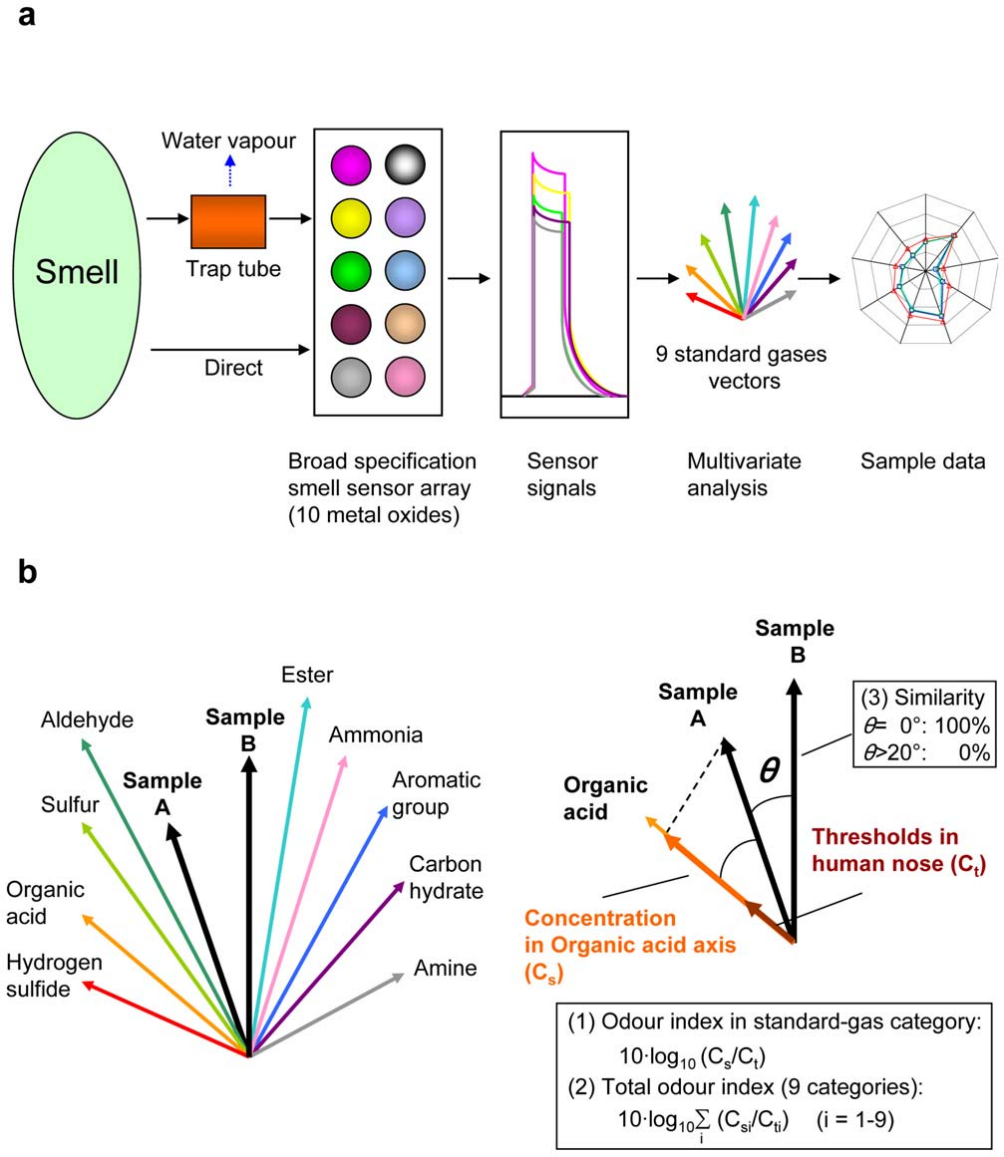
Measurement scheme and calculation methods. (a) Nine standard gases were introduced to the smell sensor array in FF-2A electronic nose system. The FF-2A used multivariate analysis to calculate the vectors of the standard gases. After yeast volatile samples were introduced and their vectors were calculated, the standard gas vectors were used to calculate the indices and similarities of yeast volatile samples. (b) Calculation method of odour index, total odour index and similarity. After all the vectors were calculated (left), the indices were calculated using virtual sample concentrations for each axis (right). Similarity was calculated using the angles between the different sample vectors.

The FF-2A electronic nose recognized odours and calculate the odour indices as described below. The device contained 10 metal oxide semiconductors sensors with different sensitivities and selectivity for different fragrant substances (Figure 1a) [22]. These sensors were standardized with 9 standard gases (hydrogen sulphide, methylmercaptan, ammonia, trimethylamine, propionic acid, butylaldehyde, butylacetate, toluene and heptane). From the sensor signals, 9 standard gas vectors were built in the space of a 10 sensor dimension. Then volatiles samples were measured and the information obtained was compared with the standard gas vectors. Finally, the data were described in terms of the standard gas categories (odour indices in standard gas categories). The calculation method for the indices was as shown in Figure 1b and as described in the Methods section mentioned below. The odour indices were quickly calculated by projecting the vectors against the standard gases categories.

Moreover, the FF-2A was installed with a trap tube for concentrating smells and removing water vapour, which can affect the measurement value (Figure 1a) [22]. This system can maintain constant sample humidity and thereby, increase the reproducibility of the measurements. Depending on the aim of the analysis, use of this trap tube is optional.

In this study, we challenged FF-2A with early detection of yeast and confirmed whether the combination method, absolute-value intensity and metaphor specification, is useful for detection and discrimination of microorganisms. To determine the lowest yeast concentration detectable, we measured the volatiles from samples obtained from 10^2^ to 10^7^ cfu/ml in the Glucose-Yeast-Peptone (GYP) media. In addition, to investigate the advantage of combining intensity and specification, we tested 2 other methods, i.e. total odour index (using smell intensity only) and similarity against other samples (using smell specification only).

## Materials and Methods

### Microorganisms and Culture Conditions

Saccharomyces sp. (yeast) and Lactococcus lactis SNW-1 (lactic acid bacteria) were provided by Sanwa Norin Co. Ltd. These microorganisms were cultured in GYP media (1.0% glucose, 0.5% yeast extract, 0.5% peptone, 0.01% MgSO_4_, 0.0005% MnSO_4_, 0.0005% FeSO_4_, and 0.002% NaCl). Both microorganisms were cultured to 1.0 McFarland and then diluted to the indicated concentrations with the media.

### Measurements of Standard Gases and Volatile Samples

The electronic nose was calibrated using the 9 standard gases as described, following which 2 ml of the samples (consisting of microorganisms and medium) were collected in 2-liter PET bags filled with dry nitrogen. The bags were allowed to equilibrate for 1 h at 25°C. The headspace volatiles were collected and diluted with dry nitrogen in new 2-liter PET bags. These diluted samples were introduced into the trap tube for 60 s and then exposed to the array with pure nitrogen gas. All the samples were measured four times and the final three measurements were used for analysis. Approximately 90 min were required to obtain the first data reading.

### Calculations

The virtual concentration for each standard-gas axis was calculated by projecting the vector obtained to the axis (Figure 1b). The odour index for each category was calculated using equation (1).
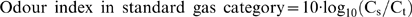
(1)


C_s_ represented the virtual concentration compared to the standard-gas axis and C_t_ was used to describe the threshold concentration (the lowest detectable concentration) of the standard gas by the human nose.

The total odour index, or the smell intensity as a whole, was calculated from the summation of the 9 standard gas intensities as in equation (2).
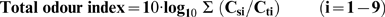
(2)


The similarity was calculated using the angles between the sample vectors. For this calculation we used the following criteria; θ = 0°, similarity 100%; θ>20°, similarity 0%.

## Results

### Analyses with Absolute-Value Intensity and Metaphor Expression

**Figure 2:**
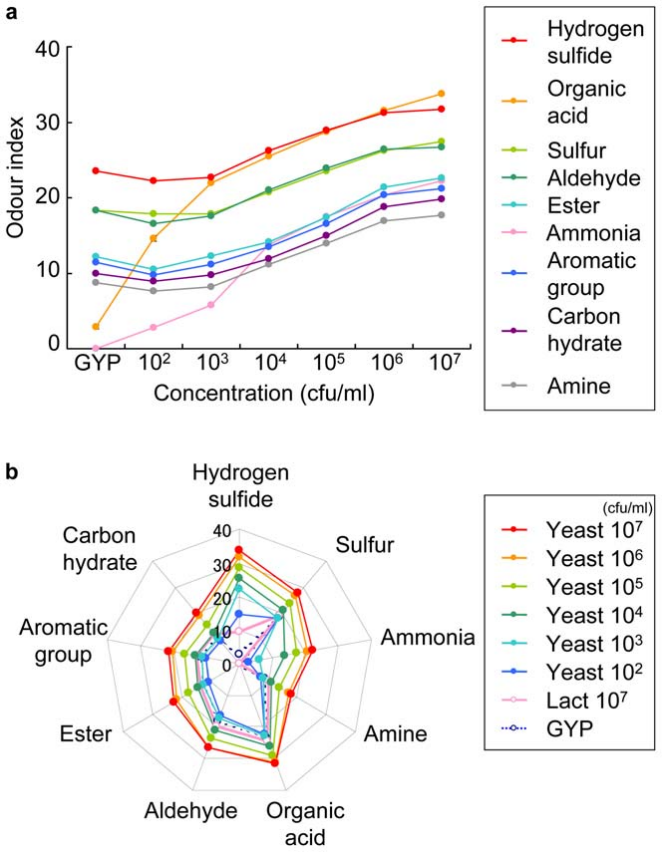
Odour index changes generated by yeast volatiles determined by 9 standard gases categories. The graph (a) and radar chart (b) depict the values calculated by the odour index expressed as mean±standard deviation (n = 3). GYP indicates the GYP control medium. Lact 10^7^ indicates the volatile sample from 10^7^ cfu/ml of lactic acid bacterium in the GYP medium (bacterium control).

Using the combination method, we showed the odour indices of the yeast volatiles in terms of the 9 standard gases categories (Figure 2). In the control media, organic acid was the highest category, and hydrogen sulphide and ammonia constituted the lower categories (odour index of the GYP media: organic acid, 23.5; hydrogen sulphide, 2.87; ammonia, 0.00). Over 10^2^ cfu/ml, the indices obtained for the lower categories, hydrogen sulphide and ammonia, increased with concentration (Figure 2a). Over 10^4^ cfu/ml, the indices for all the categories were higher than those obtained using the control media. These data suggested that our electronic nose only required 10^2^ cfu/ml to detect yeast in culture media using our chosen standard categories.

In Figure 2b, we showed the volatile data in the form of a radar chart to compare the patterns easily. We included the data obtained from using lactic acid bacteria (10^7^ cfu/ml) in this chart. Both microorganisms produced unique radar chart shapes, which were clearly different from shape generated by the control media. The analysis using the lactic acid bacteria showed that only the hydrogen sulphide category increased most when compared to the control media. Therefore, by comparing the values for the specifically increased factors, we demonstrated that we were able to discriminate yeast from lactic acid bacteria using as little as 10^2^ cfu/ml.

### Analyses with Total Odour Index

**Figure 3:**
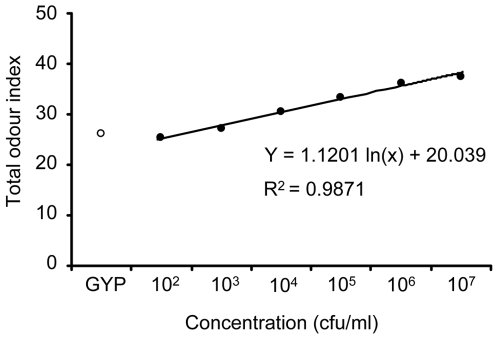
Total odour indices of yeast volatiles against concentration. These data were calculated using the smell intensities of all the volatiles in combination. The odour data are expressed as mean±standard deviation (n = 3). The approximation curve and correlation coefficient (R^2^) were calculated using Microsoft Excel 2003.

We described the data from yeast volatiles using the total odour index as the method we did with smell intensity only (Figure 3a). Different from odour index describing each gas category used above, the total odour index focuses on the smell intensity of the whole samples; all the categories were added together. From the indices obtained using this technique, we were unable to discriminate between the two microorganisms. Over 10^3^ cfu/ml, the index was higher than that obtained for the control media, and increased exponentially as Y = 1.1201 ln(x)+20.039, R^2^ = 0.9871. Although the total odour index were unable to provide us with details related to smell specification, the index can be useful for estimating the number of yeast over 10^3^ cfu/ml.

### Analyses with Similarity

**Figure 4:**
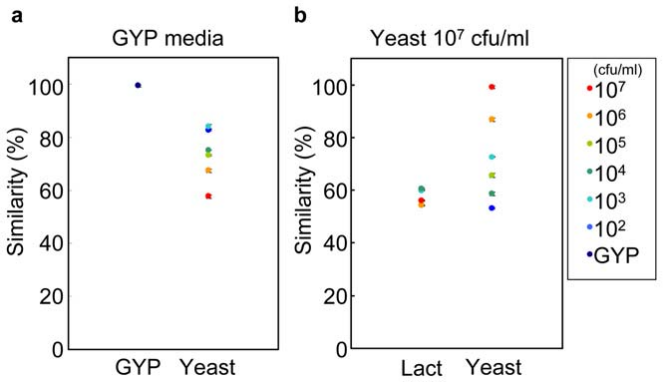
Similarities of yeast volatiles from 10^2^ to 10^7^ cfu/ml. The yeast volatiles were compared to the GYP control media (a) and yeast volatiles generated by 10^7^ cfu/ml (b). The similarities were calculated using only smell specification. The data are expressed as mean±standard deviation (n = 3). Lact indicates the volatile samples collected from 10^2^ to 10^7^ cfu/ml lactic acid bacteria (bacterium control).

Figure 4 showed the similarity of yeast volatiles compared to the GYP media alone (a), and against high concentration of yeast (b) in an attempt to analyze the samples using smell specifications only. The similarity between samples was calculated using the angles only between two axes (not including intensity information; Figure 1b). In our previous study, we learned that when the angle is 0°, our nose cannot distinguish between the 2 smells and perceives them as a single smell; this similarity of the 2 smells was described as 100% by the electronic nose. In contrast, when the angle is over 20°, we can practically distinguish between the 2 smells and this similarity was described as 0% by the device [21].

The similarities of the yeast volatiles obtained from 10^2^ to 10^7^ cfu/ml compared to the GYP media alone showed that they were all less than 84% and therefore, clearly different from the GYP media (99.3%; Figure 4a). Therefore, we can use this method to detect yeast in the GYP media. On the other hand, the similarities of the yeast volatiles from 10^2^ to 10^6^ cfu/ml compared to those from 10^7^ cfu/ml ranged from 86% to 53% (Figure 4b). With regard to the lactic acid bacteria, the similarities of the bacterial volatiles from 10^2^ to 10^7^ cfu/ml ranged from 57% to 64%. Over 10^5^ cfu/ml, the similarities of yeast volatiles were higher than the range of the lactic acid bacteria. These results suggested that we would be unable to differentiate between yeast and lactic acid bacteria using smell specification only, when the bacterial concentration was less than 10^5^ cfu/ml. The smell from the GYP media may mask the differences of these volatile specifications.

## Discussion

In this paper, we challenged early detection of yeast and compared the three methods to detect and discriminate yeast cultures using an electronic nose. The combination method that uses absolute-value intensity and metaphor specification, was extremely sensitive and could detect and discriminate yeast at the same time. Moreover, approximately 90 min were required from the sample collection to the first data reading. This shorter time analysis may help fresh food administration especially. Meanwhile, using the total odour index and similarity alone proved to be highly concentration dependent and could detect some differences. However, we could not discriminate between yeast and lactic acid bacteria using solo methods. We should combine the methods, smell intensity and specification, to detect and discriminate microorganisms at the same time.

Absolute-value smell will help record and compare smells in the development of food and drink products. For example, in the flavour of cheeses and other fermented foods, in addition to consistency and quality, there is a growing consumer demand for a larger diversity [2]. In the wine, Sauvignon Blanc, volatile thiol group are of particular importance to the varietal character, imparting passionfruit, grapefruit, in high concentrations, sweaty or cat's urine aromas [23]. Our methods will help record smells in absolute value. Strong working relationships with tasters or sommeliers may improve the quality of products and create diversity.

Furthermore, the absolute-value smell will compare the data from an electronic nose with the data from other electronic noses. The data from current electronic noses are relative values, since they have different sensors in each nose. The concept of absolute value smell is applicable to other electronic noses and will help gather data and build data bases.

For more useful information, selection of appropriate standard gasses remains as one of the key issues to be clarified. In this study, we selected the gasses from odorants related to environment offensive odours. In order to detect odorant from microorganisms sharply, GC or GC/MS data in past and current odour studies will support the selection.

Although we examined only yeast and lactic acid bacteria in this report, if scientists continue to collect smell data from different microorganisms using these methods, the resulting database will undoubtedly prove helpful in improving the safety and quality control of foods and drink. To compare and assemble such smell data, we believe that the key is using absolute values and propose the use of our combination method for the measurement of smells to assemble these databases.
